# An Optimal Seed Based Compression Algorithm for DNA Sequences

**DOI:** 10.1155/2016/3528406

**Published:** 2016-07-31

**Authors:** Pamela Vinitha Eric, Gopakumar Gopalakrishnan, Muralikrishnan Karunakaran

**Affiliations:** ^1^Department of Information Science and Engineering, Rajiv Gandhi Institute of Technology, Bangalore 560032, India; ^2^Department of Computer Science and Engineering, National Institute of Technology Calicut, Kerala 673601, India

## Abstract

This paper proposes a seed based lossless compression algorithm to compress a DNA sequence which uses a substitution method that is similar to the LempelZiv compression scheme. The proposed method exploits the repetition structures that are inherent in DNA sequences by creating an offline dictionary which contains all such repeats along with the details of mismatches. By ensuring that only promising mismatches are allowed, the method achieves a compression ratio that is at par or better than the existing lossless DNA sequence compression algorithms.

## 1. Introduction

There is an exponential increase in the amount of DNA being sequenced, thus leading to problems in storage, comprehension, and transmission. The cost of storage has been reducing dramatically in the past few years, but the exponential growth in the amount of DNA being sequenced leads to tremendous increase in the amount of data that needs to be stored online thereby making storage one of the biggest cost elements. Another challenge faced is how to make sense out of this huge mass of data. With whole genomes, we now have to deal with millions or billions of base pairs. When we have a database of such genomes, as is typically the case, the problem becomes even more compounding. Thus, new and more effective techniques are needed for the compression of biological sequence data, particularly DNA sequences.

DNA sequences are expected to be nonrandom and hence it is possible to remove redundancy, resulting in compression. It is estimated that more than 50% of the human genome is repeat DNA [1]. Compression will solve the issues related to storage and also improve the understanding of these sequences. Chen et al. [2] showed that compressibility is a good measurement of relatedness between sequences and can be effectively used in sequence alignment and evolutionary tree construction. According to Allison et al. [3] compression of DNA sequences also results in the intelligent analysis of these sequences. Compression also plays an important role in efficient sequence classification [4].

DNA sequences consist of four nucleotide bases, A (adenine), C (cytosine), G (guanine), and T (thymine), and two bits are sufficient to represent each of these nucleotide bases. Moreover the repeats found in DNA sequences are not always exact; they can be of different types like approximate, reverse, complementary, reverse complementary, and tandem. Also these repeats are long and less frequent. Traditional text compression algorithms are only effective in capturing short and frequent repeats; hence using them to compress DNA sequences often results in expansion of the same. Therefore finding all the different types of repeats in a DNA sequence and encoding them in order to achieve a good compression ratio is a challenging task.

This paper proposes a seed based algorithm which uses a substitution method that is in line with the LempelZiv [5, 6] compression scheme to compress DNA sequences. The proposed algorithm captures all the various types of repeats like exact, tandem, approximate, reverse, complemented, and reverse complemented and stores them onto an offline dictionary. These repeats are then removed from the original sequence to form the final parsed sequence. The offline dictionary along with the final parsed sequence forms the compressed sequence. Mismatches that give good compression gain are tolerated and recorded along with the repeat substrings in the offline dictionary.

This paper is organized as follows.  Section 2 reviews the various DNA compression algorithms.  Section 3 describes the proposed method and Section 4 analyzes the results obtained. This is followed by conclusion in Section 5.

## 2. Related Work

Compression of biological sequences can be either horizontal or vertical as proposed by Grumbach and Tahi [7, 8]. Horizontal mode compresses a biological sequence by making use of information contained within it, like references to the substrings, whereas vertical mode takes a set of biological sequences and compresses each sequence based on the information derived from this set. Horizontal mode is of interest for the reduction of storage and transmission costs [9] and uses compression techniques like substitution, statistical, or a combination of these two [10]. Statistical compression uses a statistical model of the data, comprised of variable sized codes, and the quality of compression obtained depends on the data model [11]. Substitution or dictionary based method selects several strings of symbols that occur frequently and encodes each string as a token which is a pointer to the string in a dictionary. The dictionary itself can be static or dynamic. Compression algorithms based on LZ method use online dictionary whereas in case of methods using offline dictionary compression occurs in two passes: the first pass identifies all repeats and stores them in a dictionary and the second pass encodes these repeats as pointers to the dictionary [5, 6]. A third category of compression is the hybrid technique which makes use of a combination of substitution and statistical techniques to compress data.

Most of the compression methods available for compressing biological sequences like [2, 7, 8, 12] use substitution methods. The earliest special purpose DNA compression algorithm found in the literature is Biocompress developed by Grumbach and Tahi [7, 8]. They proposed Biocompress and Biocompress 2 which detects repeats of substrings that occurred earlier in the sequence and encodes them as length of repeat and position of previous occurrence. They also employ order 2 arithmetic coding to encode nonrepeat regions. Chen et al. [12] developed DNACompress that uses the software utility Pattern Hunter [13] to identify significant approximate repeat regions and then encodes these repeat regions by a pointer to their earlier occurrence. The nonrepeat regions are also encoded using arithmetic coding. The offline approach by Apostolico and Lonardi [14] iteratively selects repeated substrings for which encoding would gain maximum compression. A similar substitution approach is used in GenCompress by Chen et al. [2] where they concentrate on finding an optimal prefix that can be encoded economically. Here approximate repeats are exploited. Adjeroh et al. [15, 16] create an offline dictionary of short repeats and code all occurrences of a given repeat with reference to the position of that repeat in the dictionary. Cfact developed by Rivals et al. [17] constructs a suffix tree in the first pass and uses this data structure to search for the longest exact matching repeat in the second pass.

A few methods like XM, CDNA, and ARM employ statistical techniques. Expert model (XM) proposed by Cao et al. [18] uses an order 2 Markov expert and a copy expert to predict the probability of occurrence of a symbol. It also employs adaptive coding for correct or incorrect predictions. The CDNA algorithm by Loewenstern and Yianilos [19] is a pure statistical algorithm, where the probability distribution of each symbol is obtained by approximate partial matches from history. Each approximate match is with a previous subsequence having a small Hamming distance to the context preceding the symbol to be encoded. The latter ARM algorithm by Allison et al. [3] is also a pure statistical algorithm that forms the probability of a subsequence by summing the probabilities over all explanations as to how the subsequence is generated.

A method that employs hybrid technique was introduced by Korodi and Tabus [20, 21] where encoding is done by using a simple normalized maximum likelihood model for discrete regression, through reference to preceding approximate matching blocks and encoding them by a first-order context coding. In its improvement, GeNML by Korodi and Tabus [20, 21], the DNA sequence is split into fixed size blocks. The bit mask is encoded using a probability distribution estimated by the normalized maximum likelihood of similarity between the regressor and the block. Matsumoto et al. [22] use a combination of LZ [5, 6] and CTW [23]. They first identify approximate repeat regions using hash and dynamic programming and then replace these repeat regions with an offset and length. Edit operations are encoded using arithmetic coding and nonrepeat areas by an order 32 context tree weighting.

## 3. Optimal Seed Based Compression Algorithm for DNA Sequences

The proposed method consists of a seed based algorithm that identifies potentially good matches. The matching substrings so identified are later extended in both the directions, that is, to the left and right.

Let *S* be the DNA sequence to be compressed and *l* the length of the DNA sequence. *S*_*i*_ represents the *i*th symbol of the given DNA sequence, where 1 ≤ *i* ≤ *l* and *S*_*i*,*j*_ is a substring of *S* of length *k* where *k* = *j* − *i* + 1. The seed *S*_*a*,*b*_ is also a substring of *S* of length *k*. The initial seed is *S*_1,*k*_ and the first substring (*S*_*i*,*j*_) to be matched is *S*_*k*+1,*k*+*k*_. The values of (*i*, *j*) are incremented until a repeat substring is identified such that *S*_*a*,*b*_ = *S*_*i*,*j*_. If no such matching substring is encountered, (*a*, *b*) are incremented and the search is continued until *S*_*a*,*b*_ = *S*_*i*,*j*_ for some [(*a*, *b*), (*i*, *j*)], where *i* ranges from *b* + 1 to *l* − *k* and *j* from *b* + *k* to *l*. Now the length of the match *n*_0_ is initialized to *k* and *m*, the number of mismatches, is initialized to 0.

The repeat substring *S*_*i*,*j*_ and the seed *S*_*a*,*b*_ are extended and compared. The extension is done first to the left and then to the right. The length of the match *n*_0_ is incremented for each symbol matched. If a mismatch occurs while extending the repeat substring and the seed, decision regarding permitting this mismatch is made, based on the total number of mismatches until then and whether permitting this mismatch would result in a compression gain. If *m* is greater than the threshold, repeat extension in the direction in which the mismatch occurred is temporarily terminated. Extension to the left is also stopped whenever there is an overlap between the extended seed and the extended repeat substring.

Assume that the substrings *S*_*c*,*p*_ and *S*_*d*,*q*_ are the extended repeat and the extended seed so obtained. An offline dictionary, as shown in Table 1, stores the extended seed *S*_*d*,*q*_, position of occurrence of repeat *c*, length of the repeat *n*_0_, type of repeat, and the details of mismatches that have occurred if any. *S*_*c*,*p*_ is then removed from *S* and the remaining symbols of *S* are concatenated to form the next sequence *S*_*p*_. The process is repeated on sequence *S*_*p*_ until all approximate repeats of *S*_*d*,*q*_ are identified and stored in the offline dictionary. This offline dictionary is similar to the one created by Adjeroh et al. [15, 16, 25].

Finally the extended seed *S*_*d*,*q*_ is removed from *S*_*p*_ and the remaining symbols of *S*_*p*_ are concatenated to form the new sequence *S*. The position of the extended seed *S*_*d*,*q*_,  *d*, is recorded as the last entry under that seed.

**Figure 1:**
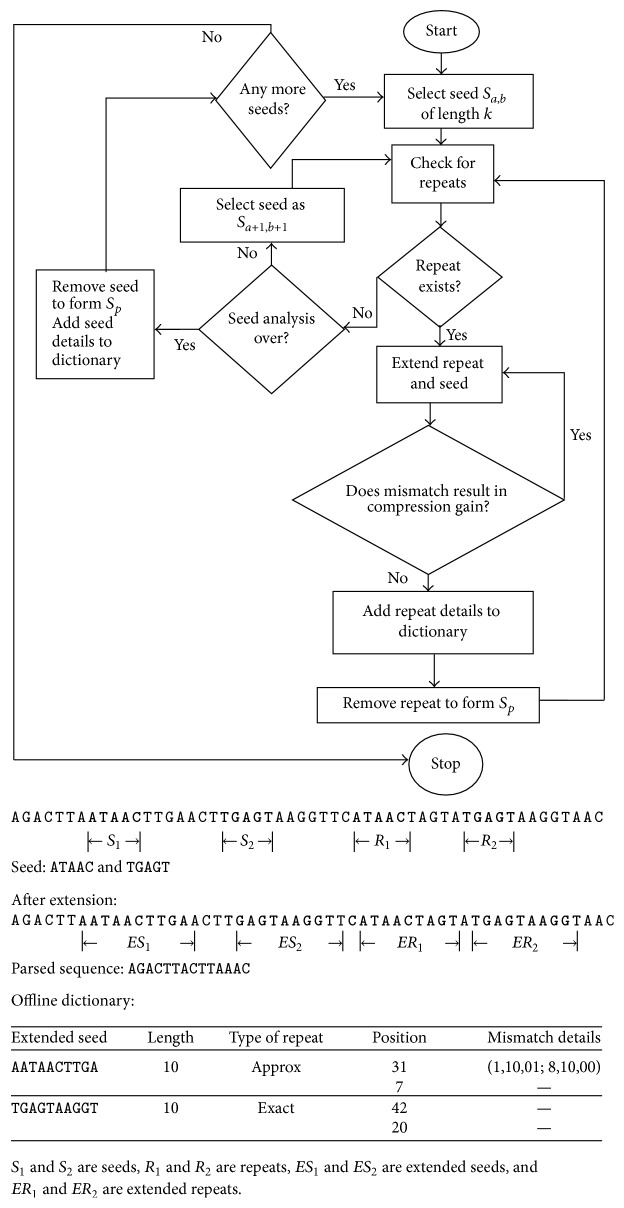
The process flow of the seed based compression method followed by an example sequence.

The above process is repeated on the new sequence *S* until all the exact and approximate repeats are identified and removed from the sequence and the remaining nonrepeat regions of the sequence are concatenated to form the final parsed sequence. This process along with an example is depicted in Figure 1. The offline dictionary along with the final parsed sequence (the original sequence from which all seeds and repeats have been removed) forms the compressed sequence. The final parsed sequence is further compressed using adaptive arithmetic coding. The extended seed entries in the offline dictionary are also encoded using arithmetic coding.

### 3.1. Encoding of Mismatches

The proposed method identifies all significant approximate repeats in the DNA sequence and stores them onto an offline dictionary. All approximate repeats have mismatches which are encoded and written onto the offline dictionary. Mismatches occur due to mutation and can be defined by any of the edit operations like insertion, deletion, or substitution of some base. The mismatch details are recorded in the table as a triple (*P*, *E*, *S*), where *P* is the position of mismatch within the extended seed, *E* the type of edit operation, and *S* the symbol to be inserted or substituted. When the edit operation is deletion, the last field of the triple may be omitted. The same representation is also used in DNACompress [12] and GenCompress [2]. The edit operations are encoded as 00 insertion, 01 deletion, and 10 substitution and the bases A, C, G, and T are encoded as 00, 01, 10, and 11, respectively. Suppose that the seed is the substring GCACTTACT and the approximate repeat found is GCACTTTCT. Here the symbol A which occurs at the 7th position in the seed has been substituted with the symbol T in the repeat. This is represented by a triple of 7 bits as (1111011).

### 3.2. Determining the Threshold

Mismatches are allowed while extending the repeats in both directions but the number of such mismatches should not exceed a predetermined threshold. When the predetermined threshold is exceeded temporarily suspend extension of seed and repeat in that direction until the length of the repeat and seed has increased to such an extent that extension of repeat in the suspended direction becomes feasible again. But any mismatch is tolerated if and only if allowing such a mismatch results in a compression gain. The threshold value is determined dynamically with respect to the length of the extended repeat. Experimental results show that the total number of mismatches allowed at any instance should never exceed log_2_⁡(*n*_0_); here (*n*_0_) is the length of the extended repeat.

### 3.3. Calculation of Compression Ratio

It takes at the most 2 bits to encode each symbol of a DNA sequence. The objective of DNA compression is to bring down the bits needed to represent each base to less than 2. In the proposed method the output comprises the offline dictionary along with the final parsed sequence and the compression ratio specifies the bits per symbol (bps) and can be calculated by the following formula [16]:

(1) Compression ratio=Cost of output sequenceLength of input sequence=Cost of dictionary+Cost of parsed sequenceLength of input sequence.

The cost of a variable is the number of bits required to represent it. The term vocabulary refers to the identified repeats without reference to their specific locations in the sequence. The size of the dictionary denoted as *n* gives the number of distinct repetitions in the dictionary. The length of the *i*th seed is denoted by *l*(*i*) and the number of repetitions of *i*th seed by *k*(*i*). If the position of the *j*th occurrence of repeat pattern *i* is given as *P*(*i*, *j*) and mismatch in the *j*th occurrence of repeat pattern *i* as *M*(*i*, *j*), then cost of positions and mismatch details can be given as

(2) ∑i=1n ∑j=1kilogPi,j+∑i=1n ∑j=1kiMi,j+∑i=1n ∑j=1kilogli+∑i=1n ∑j=1ki2.

Here log(*l*(*i*)) is the number of bits required to represent *l*(*i*) and the last term gives the number of bits needed to represent the type of repetition of each repeat.

The cost of dictionary is the sum of the cost of vocabulary and cost of positions and mismatch details:

(3) Cost of vocabulary=2∗Sum of length of extended seeds=2∗∑i=1nli.

Therefore

(4) Cost of dictionary=2∗∑i=1nli+∑i=1n ∑j=1kilog⁡Pi,j+∑i=1n ∑j=1kiMi,j+∑i=1n ∑j=1kilog⁡li+∑i=1n ∑j=1ki2.

The final parsed sequence *S*_final_ is the sequence from which all the repeats have been removed. The number of bits to represent *S*_final_ is 2*∗l*(*S*_final_). Therefore

(5) Compression ratio=2∗∑i=1nli+∑i=1n∑j=1kilogPi,j+∑i=1n∑j=1kiMi,j+∑i=1n∑j=1kilogli+∑i=1n∑j=1ki2+2∗lSfinalLength of input sequence.

## 4. Results

The seed based compression algorithm was experimentally verified on a set of DNA sequences in FASTA format as the input. The method was tested on the same standard benchmark data used in [2, 7, 12, 18, 22]. These standard sequences include human growth hormone (HUMGHCSA), human DNA sequence (HUMHDABCD), vaccinia virus Copenhagen complete genome (VACCG),* Marchantia polymorpha* mitochondrion complete genome (MPOMTCG), human beta globin region on chromosome 11 (HUMHBB),* Homo sapiens* dystrophin gene (HUMDYSTROP), and human hypoxanthine phosphoribosyltransferase gene (HUMHPRTB).

**Figure 2:**
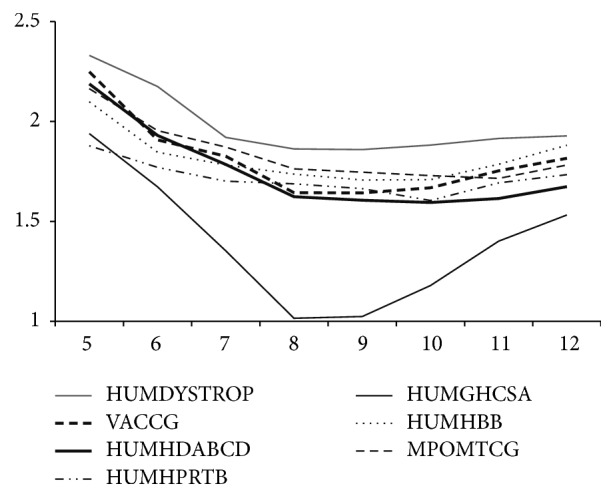
Graph comparing compression ratios against varying *k* values for different sequences; *x*-axis : “*k*” value; *y*-axis : compression ratio.

The algorithm was tested on this data set for various seed lengths “*k*,” to decide upon an optimum “*k*” value for the compression. The seed length “*k*” was varied from 5 to 11 on various runs of the data set and the best “*k*” value was inferred to be 8 as this gives better compression ratio than smaller “*k*” values. BLAST [26], being another bioinformatics local alignment search tool, also uses 11 as the standard seed length whereas SENSEI [27] uses 8 as the seed length. Also, it was noticed that even though time complexity increases when *k* was incremented further a substantial improvement in compression ratio does not occur to warrant such an increase. Thus the “*k*” value was inferred to be 8. A graph comparing compression ratios against varying *k* values for different sequences is shown in Figure 2.

**Figure 3:**
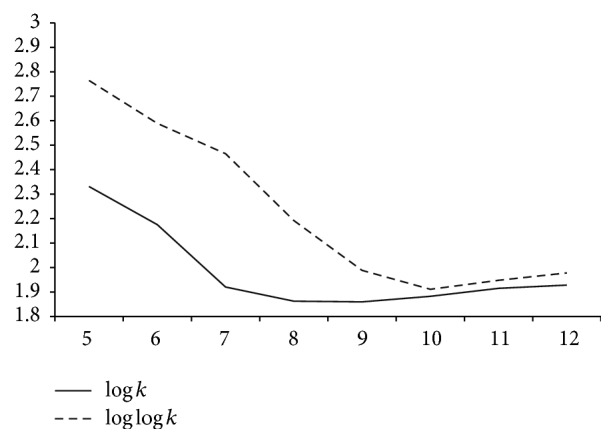
Graph comparing compression ratios of HUMDYSTROP against varying *k* values and the threshold for the number of mismatches allowed being log⁡*k* and log⁡log⁡*k*. *x*-axis : “*k*” value; *y*-axis : compression ratio.

Any mismatch is tolerated if and only if allowing such a mismatch results in a compression gain. To ensure compression gain a mismatch is allowed only if the next few characters are an exact match. The results of testing for different values showed that ensuring the next three characters are exact matches gives good compression ratio. The permitted threshold for the number of mismatches allowed was varied from log_2_⁡*k* to log_2_⁡log_2_⁡*k* and it was found that log_2_⁡*k* gives better compression. The graph depicting the compression ratio achieved when the threshold for the number of mismatches allowed is log_2_⁡*k* and log_2_⁡log_2_⁡*k* is shown in Figure 3.

The tabulated result of comparison of compression ratios of the proposed seed based method against other existing algorithms is shown in Table 2.

The execution time taken by few of the reviewed methods for the benchmark sequences was determined after executing them on a machine with a quad core processor having a clock speed of 2.60 GHz, 8 GB RAM, and 64-bit operating system. The execution time taken by the reviewed methods is given in Table 3.

*Decompression*.  Algorithm 1 was implemented and the result of decompression was verified to ensure that the compression method proposed is indeed lossless.

## 5. Conclusion

A substitutional compression algorithm for DNA sequence is proposed. On extensive testing, the optimum seed length for the method was decided to be 8. As seen from the results, it is observed that the proposed method performs with compression ratios comparable to the existing algorithms and even better for a few standard sequences. Further the speed of execution can be improved by incorporating any known methods of string comparison like suffix trees or bitwise XOR operation as used in SENSEI [27] in the initial phase while looking for exact seed matches.

## Figures and Tables

**Algorithm 1:**
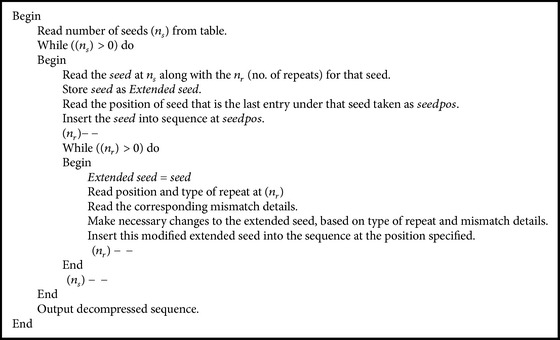
Decompression algorithm.

**Table 1:** Structure of the offline dictionary.

Extended seed	Type of repeat	Position of repeat	Length	Mismatch details
AATAACTTG	Approx	5	9	
	Reverse	20	9	(1 10 01, 8 10 00)

AACTTG	Reverse	36	6	
	Approx	73	7	(4 01 10)

**Table 2:** Comparison of compression ratios of the proposed method against existing methods [2, 8, 12, 18, 19, 21, 22, 24].

Sequence	Length	CDNA	GeMNL	Bioc	CTW + LZ	GenC	DNAC	DNAP	XM	Proposed seed based method
HUMDYSTROP	38,770	1.93	1.9085	1.9262	1.9175	1.9231	1.9116	1.9088	1.9031	1.8624
HUMGHCSA	66,496	0.95	1.0089	1.3072	1.0972	1.0969	1.0272	1.639	0.9828	1.0156
HUMHBB	73,308	1.77	—	1.8800	1.8082	1.8204	1.7897	1.7771	1.7513	1.7364
HUMHDABCD	58,863	1.67	1.7059	1.8770	1.8218	1.8192	1.7951	1.7394	1.6671	1.6237
HUMHPRTB	56,832	1.72	1.7639	1.9066	1.8433	1.8466	1.8165	1.7886	1.7361	1.688
MPOMTCG	1,86,609	1.87	1.8822	1.9378	1.9000	1.9058	1.8920	1.8932	1.8768	1.763
VACCG	1,91,735	1.81	1.7644	1.7614	1.7616	1.7614	1.7580	1.7583	1.6749	1.6434

**Table 3:** Time taken for execution.

Sequence	Length	DNACompress (sec)	GenCompress (sec)	Time taken by seed based method (sec)
HUMDYSTROP	38,770	0.125	0:00:45	1.5
HUMGHCSA	66,496	0.094	874	2.5
HUMHBB	73,308	0.125	NA	2.8
HUMHDABCD	58,863	0.125	104	2.2
HUMHPRTB	56,832	0.124	90	2
MPOMTCG	1,86,609	0.124	781	3.5
VACCG	1,91,735	0.219	1239	4
